# Supplementary material for: Simultaneous enhanced efficiency and thermal stability in organic solar cells from a polymer acceptor additive
Source: Nat Commun. 2020 Mar 5;11:1218. doi: 10.1038/s41467-020-14926-5 (PMC7057953; doi:10.1038/s41467-020-14926-5)
Supplement: Supplementary file 1 — Supplementary Information [file 41467_2020_14926_MOESM1_ESM.pdf]

Supplementary Information

for

Simultaneous Enhanced Efficiency and Thermal Stability in Organic Solar Cells From A Polymer Acceptor Additive

Wenyan Yang,[1 #] *Zhenghui Luo,*[2 #] *Rui Sun,*[1] *Jie Guo,*[1] *Tao Wang,*[1] *Yao Wu,*[1] *Wei Wang,*[1] *Jing Guo,*[1] *Qiang Wu,*[1] *Mumin Shi,*[1] *Hongneng Li,*[1] *Chuluo Yang,*[2] *Jie Min*[1,3,4]*

[1]W. Yang, R. Sun, J. Guo, T. Wang, Y. Wu, W. Wang, J. Guo, Q. Wu, M. Shi, H. Li, Prof. J. Min

The Institute for Advanced Studies, Wuhan University, Wuhan 430072, China

E-mail: min.jie@whu.edu.cn

[2]Z. Luo, Prof. C. Yang

Hubei Key Lab on Organic and Polymeric Optoelectronic Materials, Department of Chemistry, Wuhan University, Wuhan 430072, China

[3]Beijing National Laboratory for Molecular Sciences, Beijing, 100190 China

[4]Key Laboratory of Materials Processing and Mold (Zhengzhou University), Ministry of Education, Zhengzhou, 450002 China

[#]The first two authors contributed equally to this paper.

Supplementary Information

Supplementary Figures

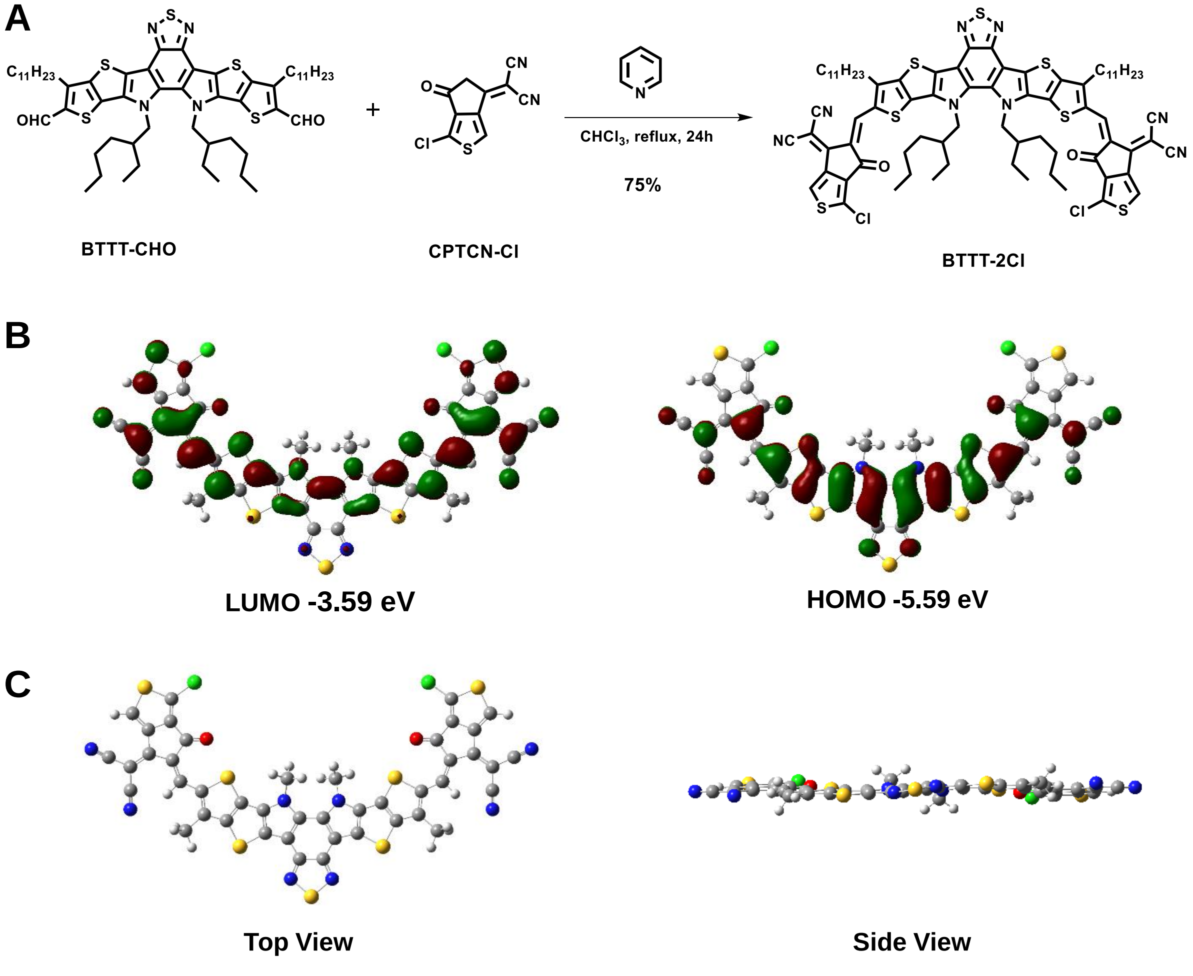

Supplementary Figure 1. (A) Synthetic route of non-fullerene acceptor (NFA) BTTT-2Cl. (B) LUMO and HOMO energy levels as well as (C) molecular geometry of BTTT-2Cl calculated by DFT/B3LYP/6-31G(d, p) with methyl groups in replacing alkyl substituents to simplify the calculations.

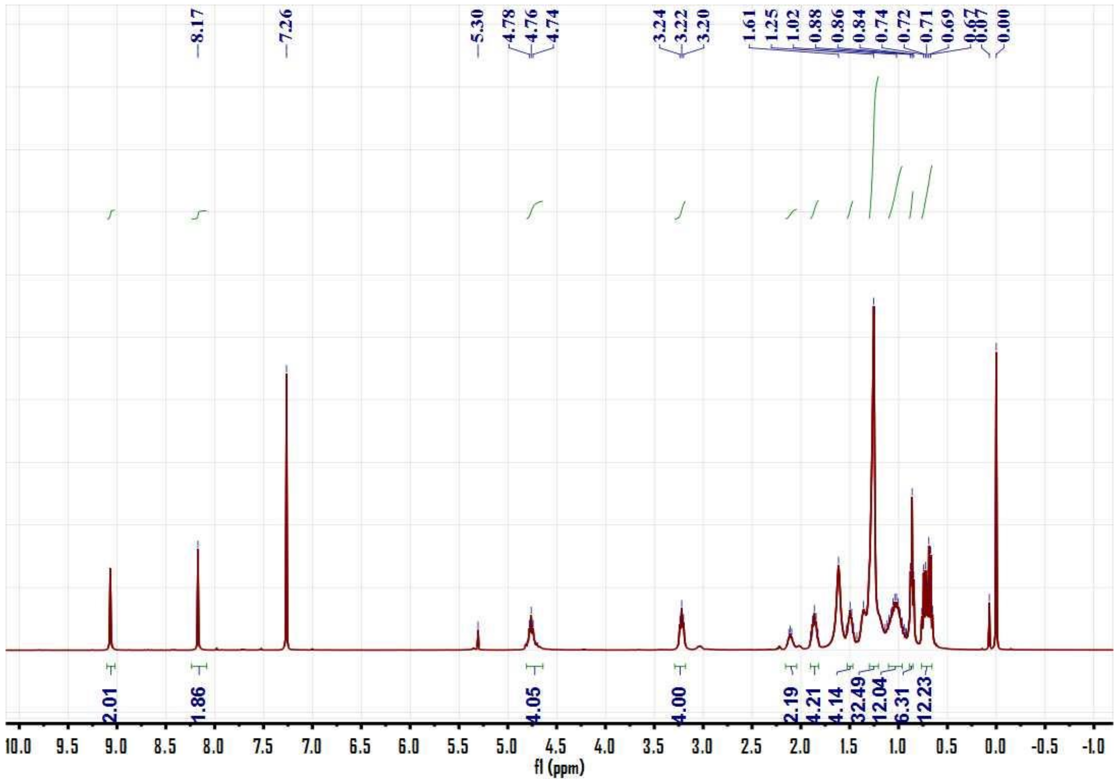

Supplementary Figure 2. ^{1}H NMR spectrum of BTTT-2Cl.

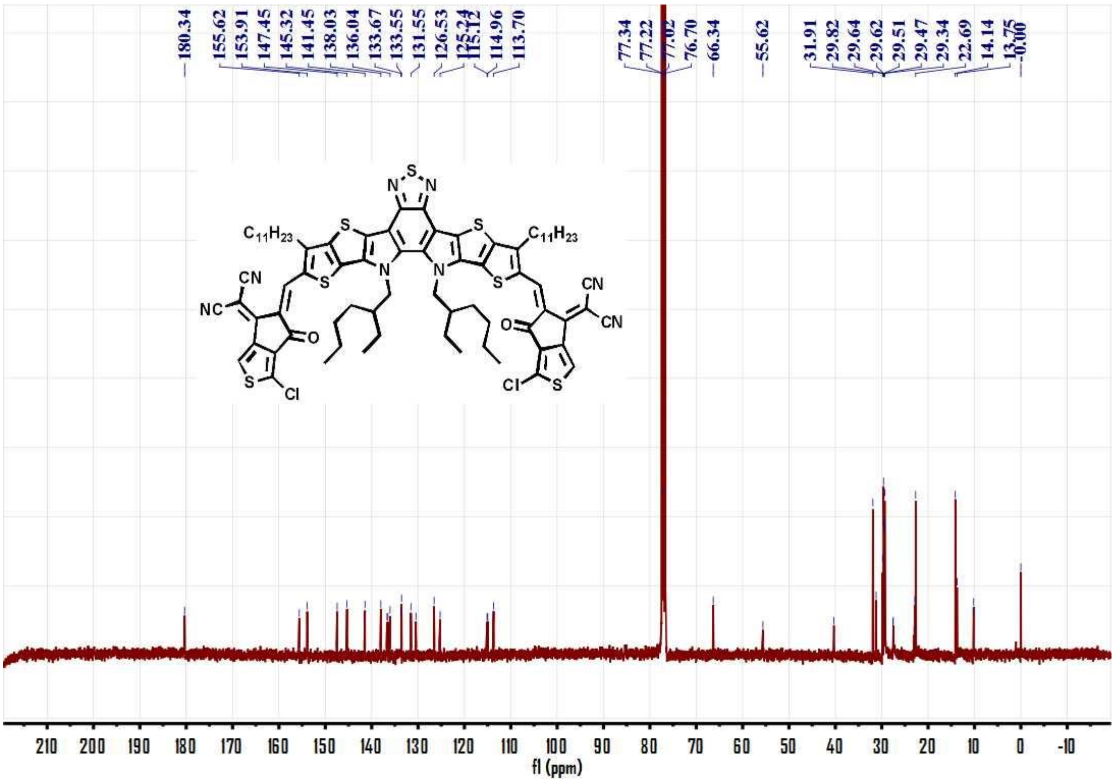

Supplementary Figure 3. ^{13}C NMR spectrum of BTTT-2Cl.

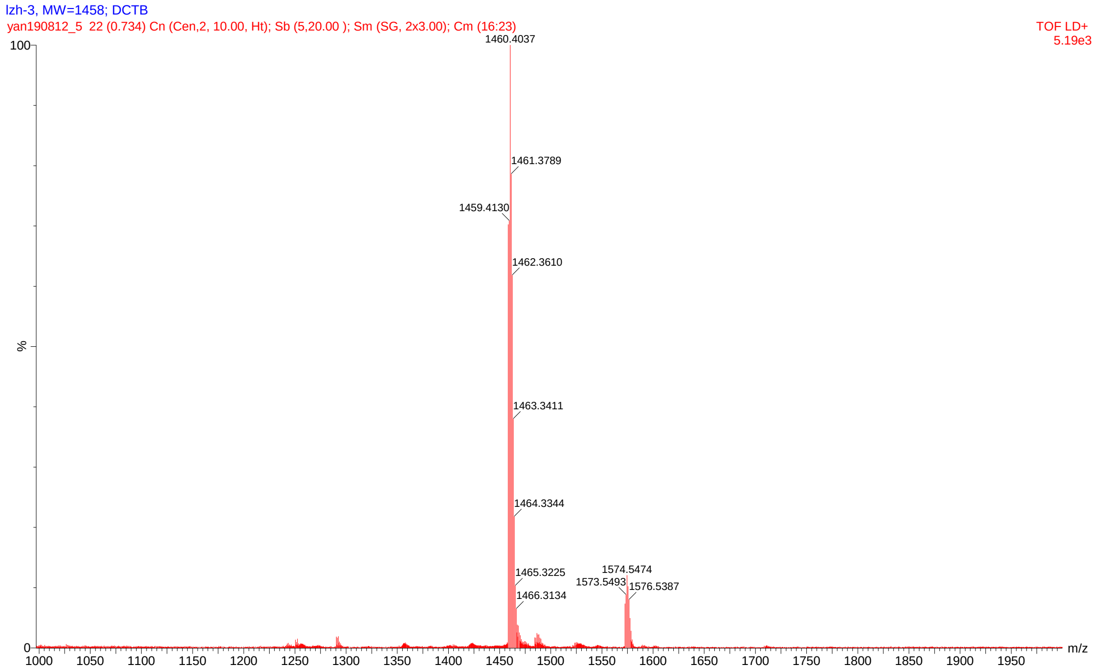

Supplementary Figure 4. MS spectrum (MALDI-TOF) of BTTT-2Cl.

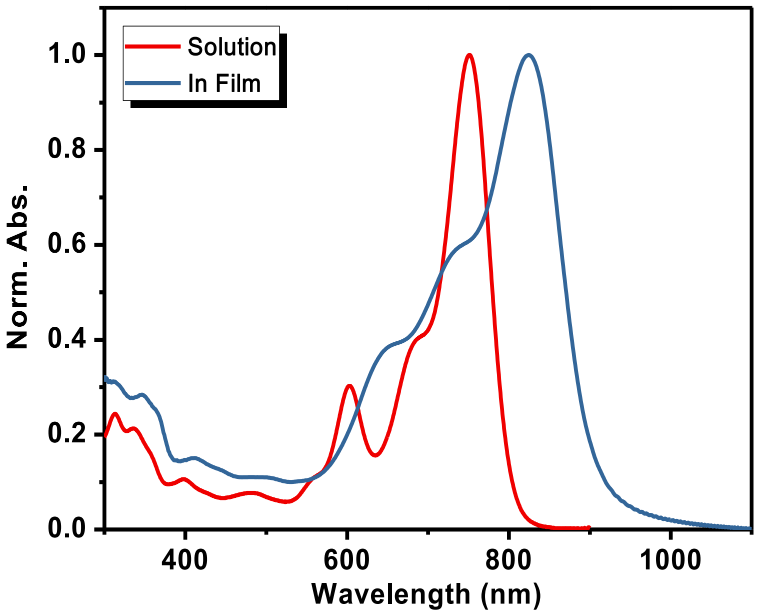

Supplementary Figure 5. UV-vis absorption spectra of BTTT-2Cl in solution and in the solid state.

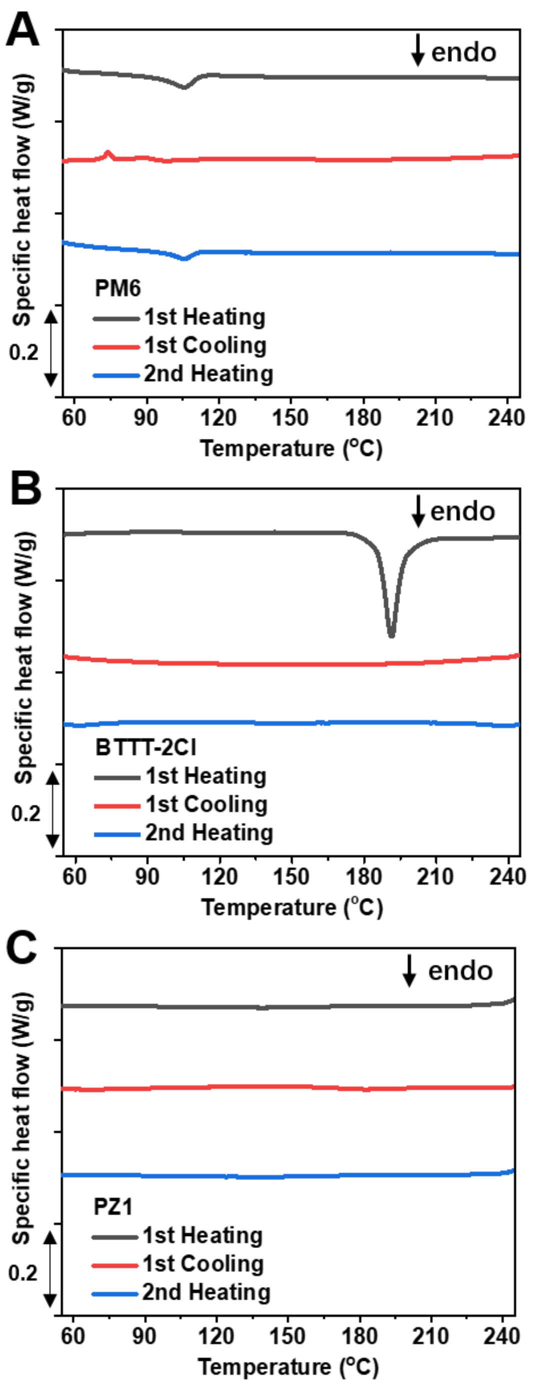

Supplementary Figure 6. DSC tests of (A) PM6, (B) BTTT-2Cl and (C) PZ1 under the nitrogen flow.

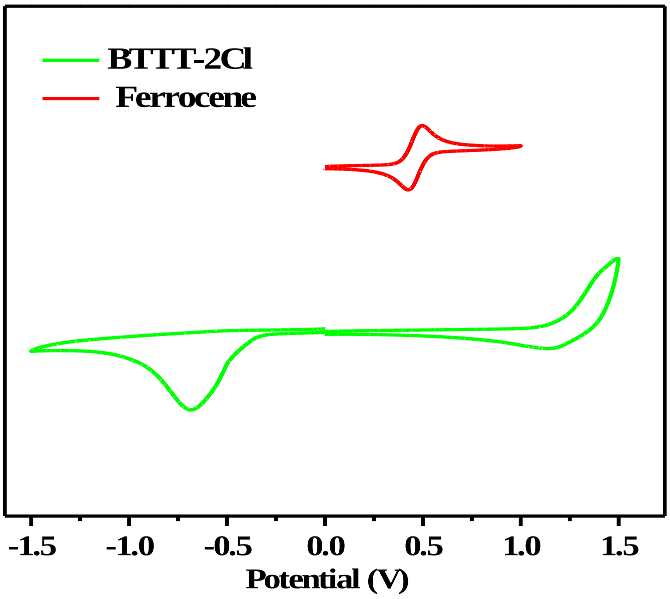

Supplementary Figure 7. Cyclic voltammogram (CV) curve of BTTT-2Cl on Pt electrode in 0.1M Bu4NPF6, CH_3CN solution, the insert figure (red line) shows the CV of ferrocene/ferrocenium (Fc/Fc+) couple used as an internal reference.

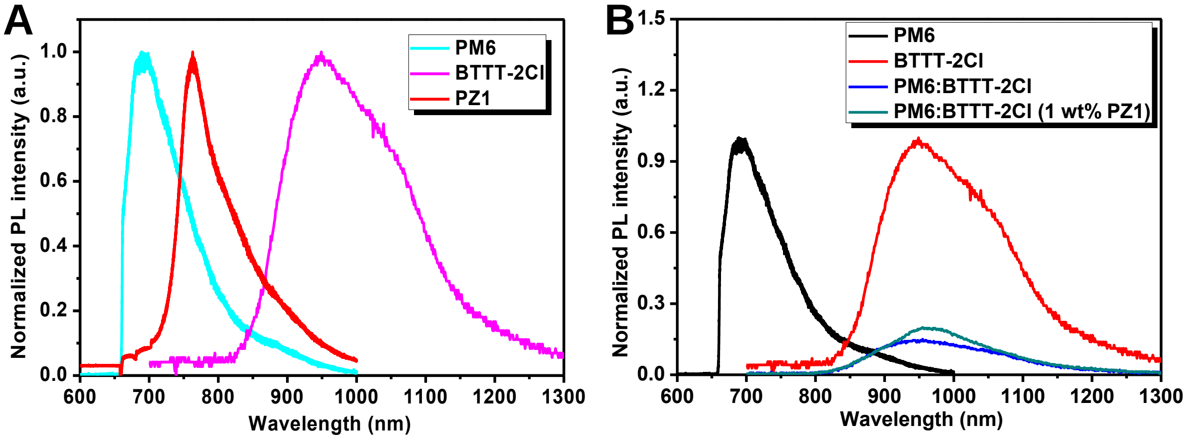

Supplementary Figure 8. (A) Photoluminescence (PL) spectra of the pure PM6, BTTT-2Cl and PZ1 films. (B) PL quenching spectra of pure PM6, BTTT-2Cl and PM6:BTTT-2Cl blend films without and with 1 wt% PZ1. Excitation of the films was at 639 nm with the exception of the PL spectra of PM6 film. The PL quenching efficiency of the PM6:BTTT-2Cl blend is estimated to be about 80%. Interestingly, the PL intensity of PM6:BTTT-2Cl blend film increases slightly with adding 1 wt% PZ1, suggesting that the interface area between PM6 and BTTT-2Cl decreases. This is consistent with the AFM and GIWAXS data as shown in Fig. 1d and Supplementary Figure 9, and confirms that the growth of pure domains closer to the exciton diffusion length, allows more efficient extraction of the photogenerated charge carriers.

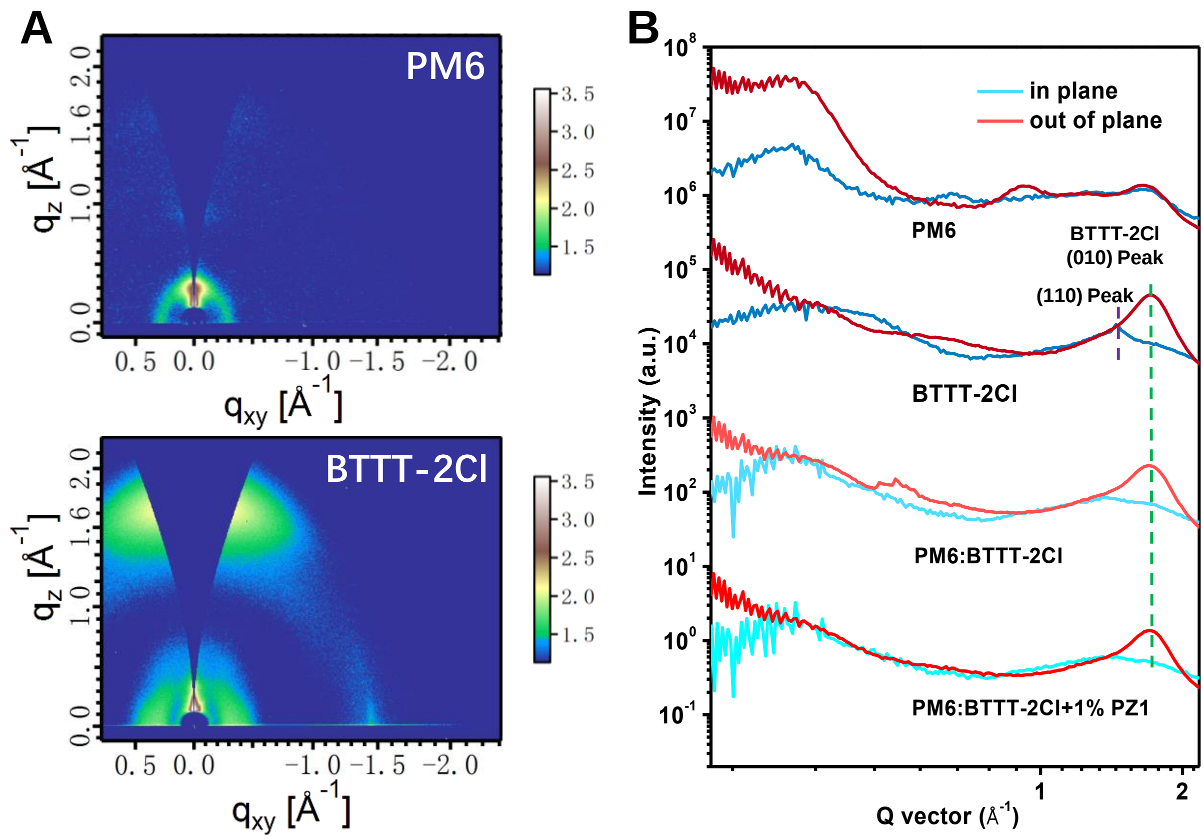

Supplementary Figure 9. (A) 2D-GIWAXS patterns of pristine PM6 and BTTT-2Cl films. (B) The 1D GIWAXS line curves of the corresponding pristine films and blend systems with respect to the in-plane direction and out-of-plane direction. The data was acquired at the critical incident angle of 0.13°.

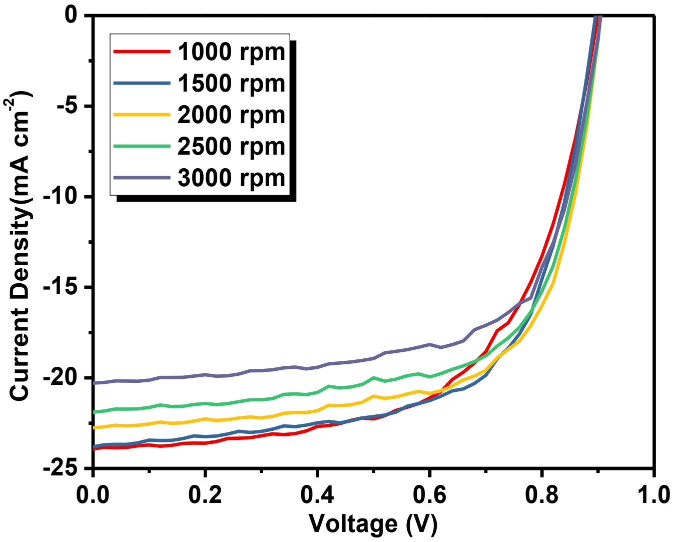

Supplementary Figure 10. *J-V* curves of PM6:BTTT-2Cl devices with different spin-coating speeds measured under one sun illumination.

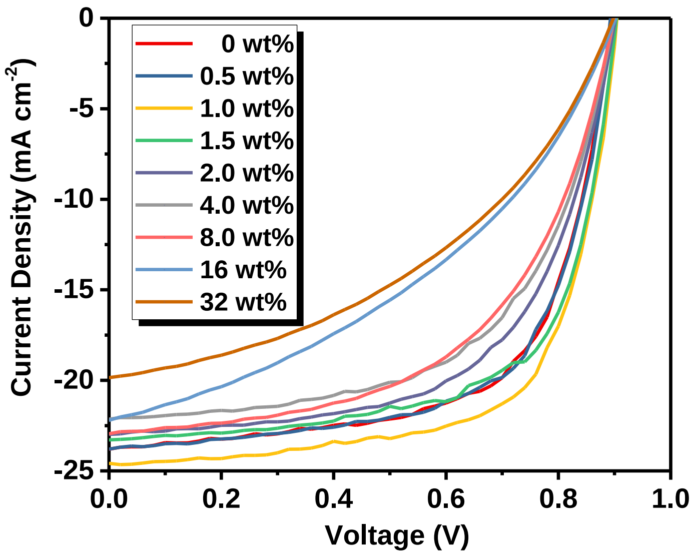

Supplementary Figure 11. *J-V* curves of PM6:BTTT-2Cl devices with different PZ1 concentrations, measured under one sun illumination.

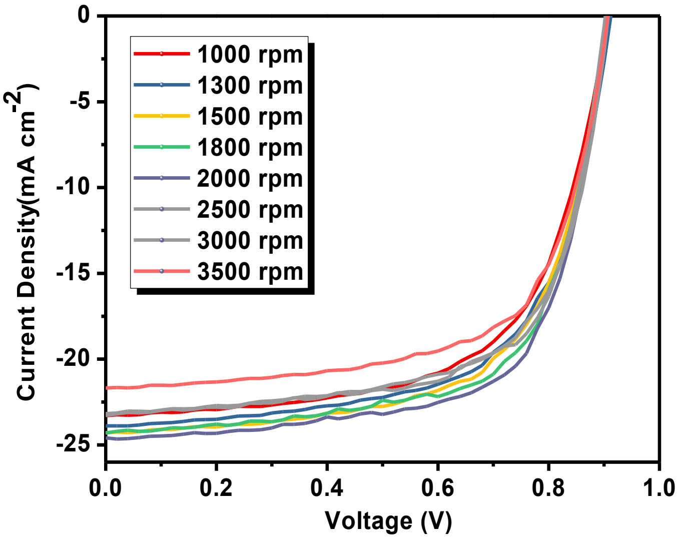

Supplementary Figure 12. *J-V* curves of 1 wt% PZ1-incorporated PM6:BTTT-2Cl devices with different spin-coating speeds measured under one sun illumination.

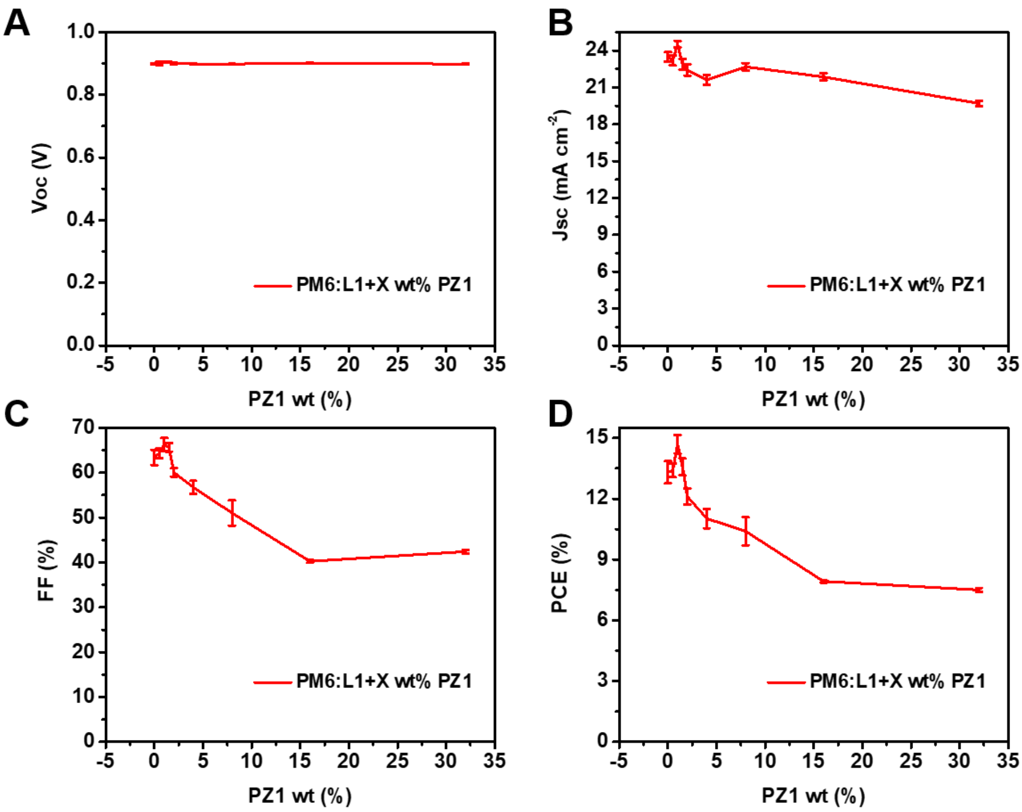

Supplementary Figure 13. Photovoltaic parameters ((A) V_{oc}, (B) J_{sc}, (C) FF and (D) PCE) of PM6:BTTT-2Cl organic solar cells as a function of PZ1 concentrations, measured under one sun illumination.

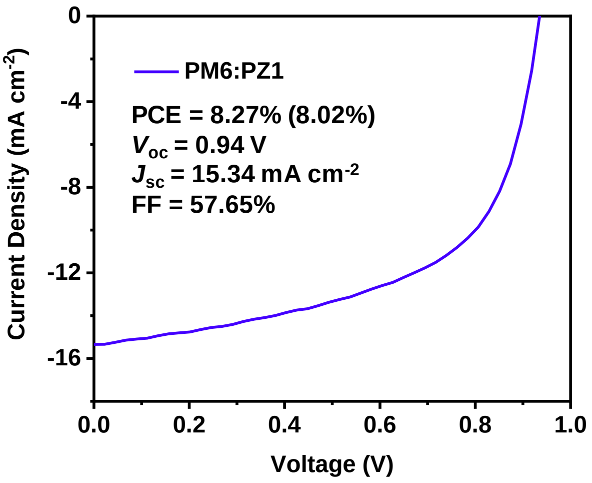

Supplementary Figure 14. *J-V* curves of PM6:PZ1 (1.75:1, wt%) devices measured under one sun illumination. The average PCE value of 8.02% was calculated by eight devices.

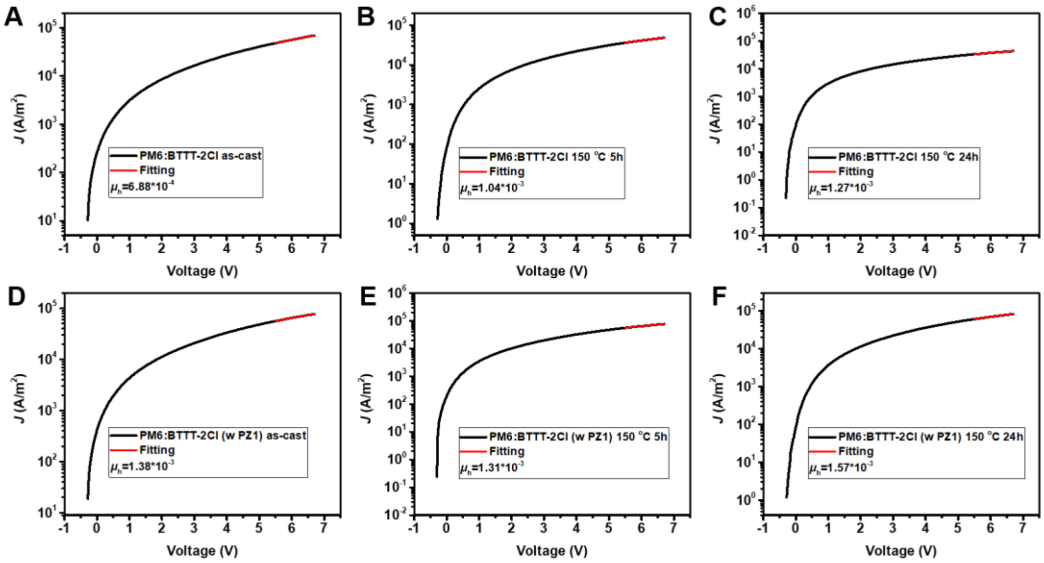

Supplementary Figure 15. The dark *J-V* characteristics of PM6:BTTT-2Cl films (A) without heating, with 150 °C for (B) 5 h and (C) 24 h based hole-only devices, respectively; The dark *J-V* characteristics of 1 wt% PZ1-incorporated PM6:BTTT-2Cl films (D) without heating, with 150 °C for (E) 5 h and (F) 24 h based hole-only devices, respectively. The red lines represent the best fitting using the SCLC model.

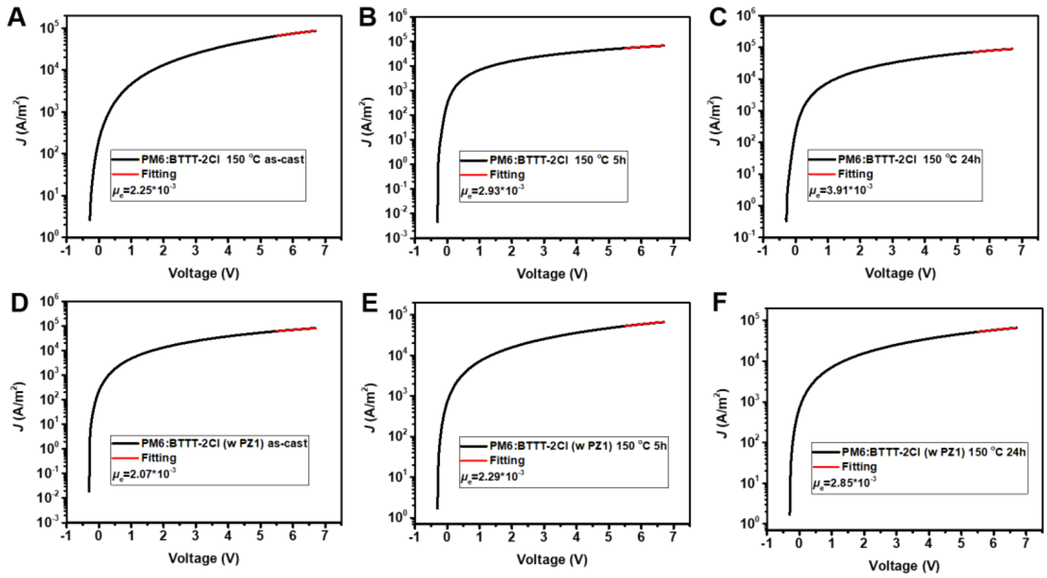

Supplementary Figure 16. The dark *J-V* characteristics of PM6:BTTT-2Cl films (A) without heating, with 150 °C for (B) 5 h and (C) 24 h based electron-only devices, respectively; The dark *J-V* characteristics of 1 wt% PZ1-incorporated PM6:BTTT-2Cl films (D) without heating, with 150 °C for (E) 5 h and (F) 24 h based electron-only devices, respectively. The red lines represent the best fitting using the SCLC model.

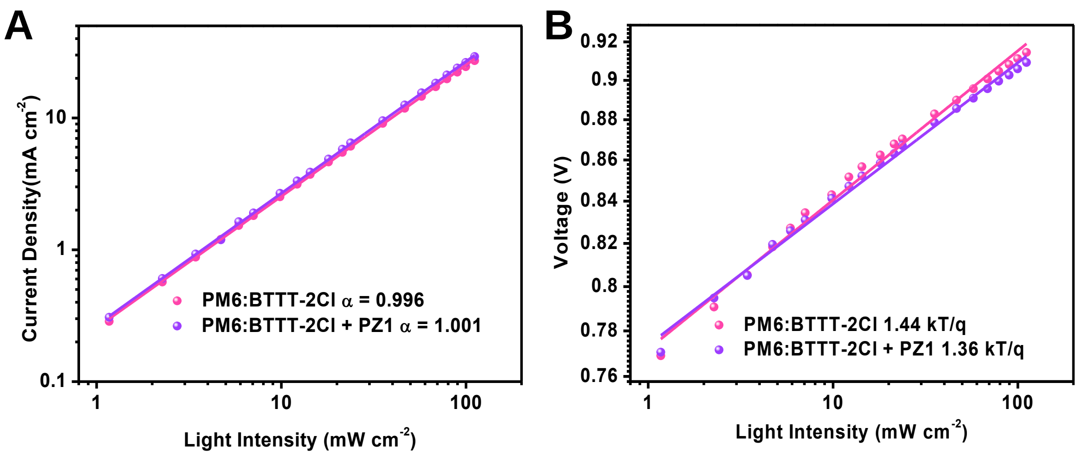

Supplementary Figure 17. (A) Current density and (B) voltage against light intensity of the relevant devices. Intensities are corrected for AM1.5G spectral mismatch.

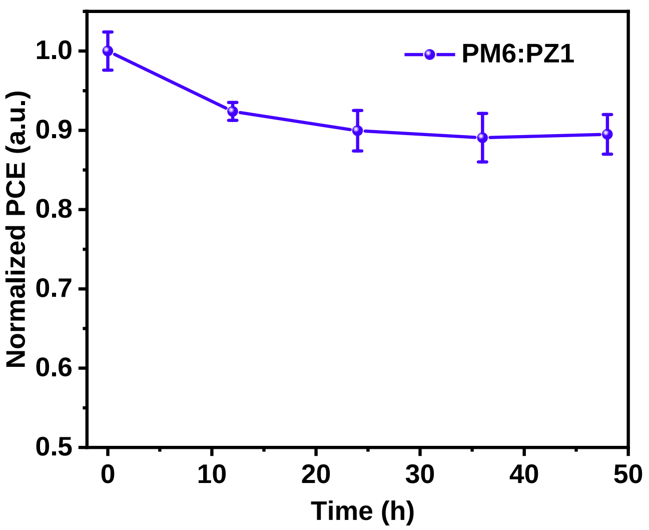

Supplementary Figure 18. Normalized PCE of the PM6:PZ1 blend as a function of heating time at 150 °C.

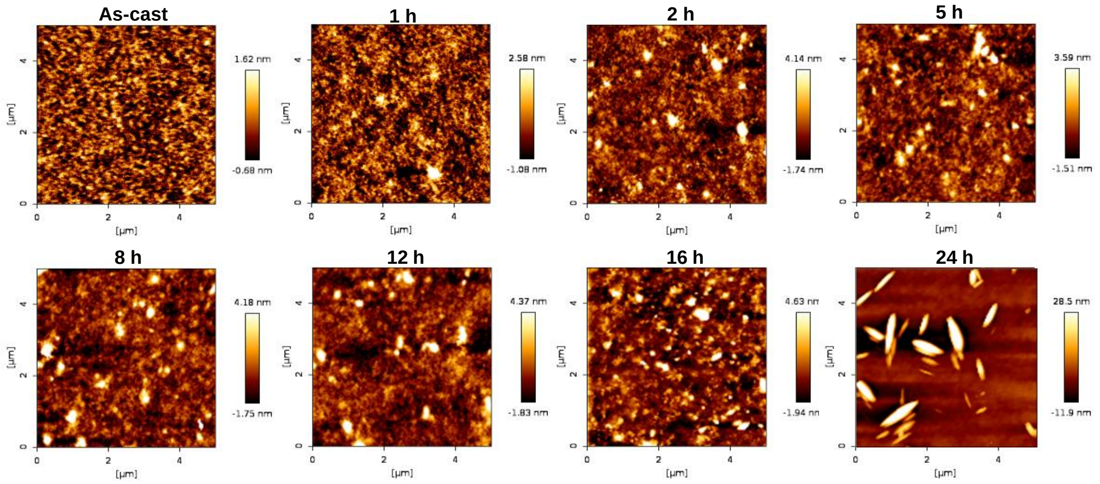

Supplementary Figure 19. AFM topography images of PM6:BTTT-2Cl blend films as a function of annealing time, including, 0 h, 1h, 2h, 5h, 8h, 12 h, 16 h and 24h, at 150 °C.

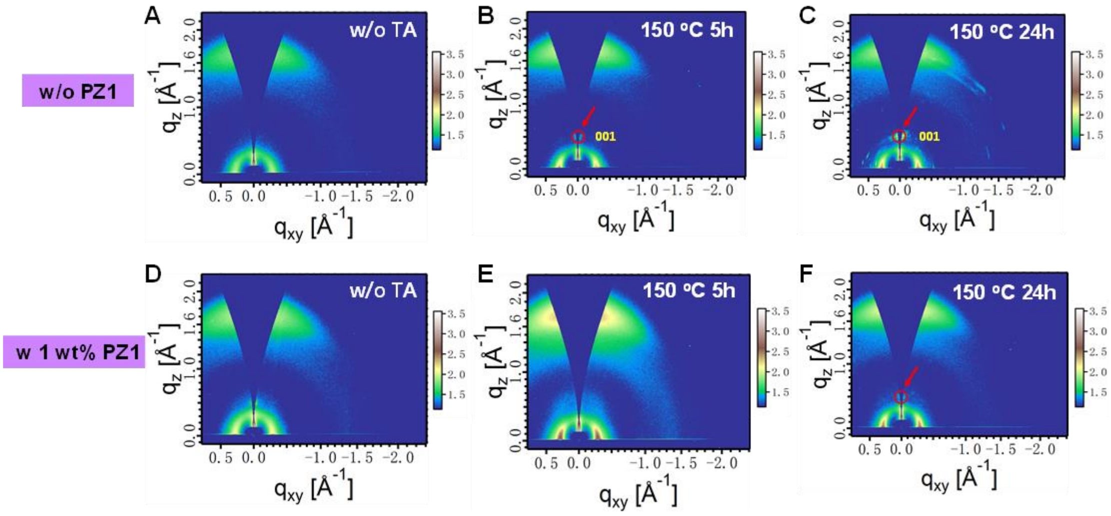

Supplementary Figure 20. 2D-GIWAXS patterns of PM6:BTTT-2Cl films (A) without heating, with 150 °C for (B) 5 h and (C) 24 h, respectively; 2D-GIWAXS patterns of 1 wt% PZ1-incorporated PM6:BTTT-2Cl films (D) without heating, with 150 °C for (E) 5 h and (F) 24 h, respectively. The data was acquired at the critical incident angle of 0.13°.

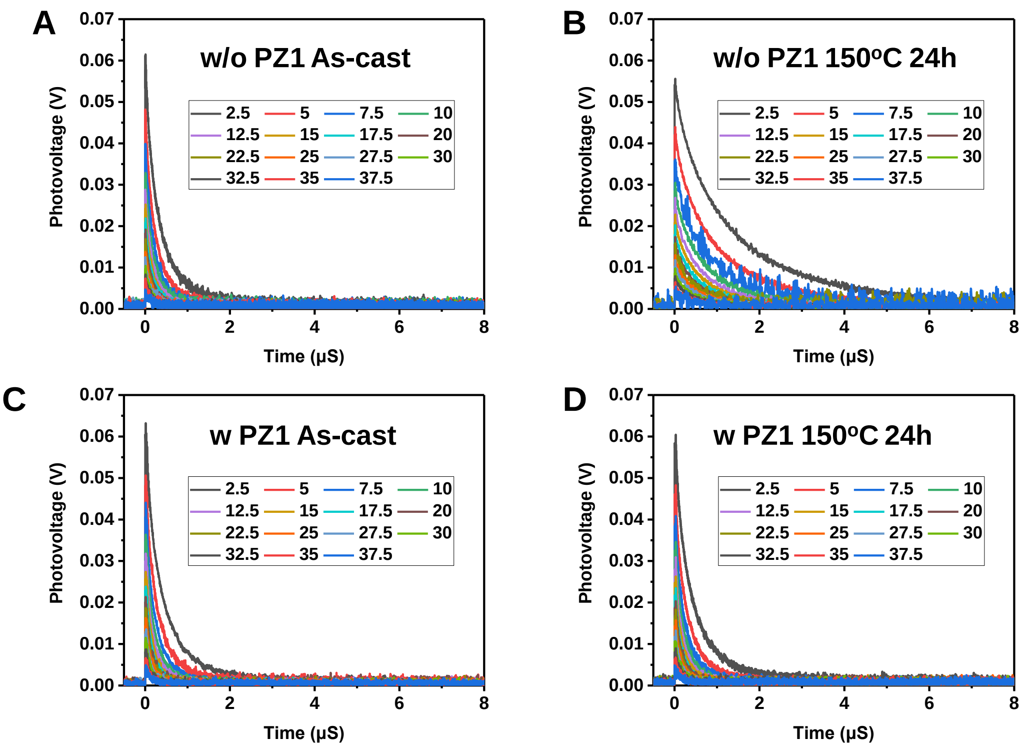

Supplementary Figure 21. TPV measurements on PM6:BTTT-2Cl films (A) without heating and (B) with 150 °C for 24 h based devices for light intensities of 0.15 to 2.50 sun; TPV measurements on 1 wt% PZ1-incorporated PM6:BTTT-2Cl films (C) without heating and (D) with 150 °C for 24 h based devices.

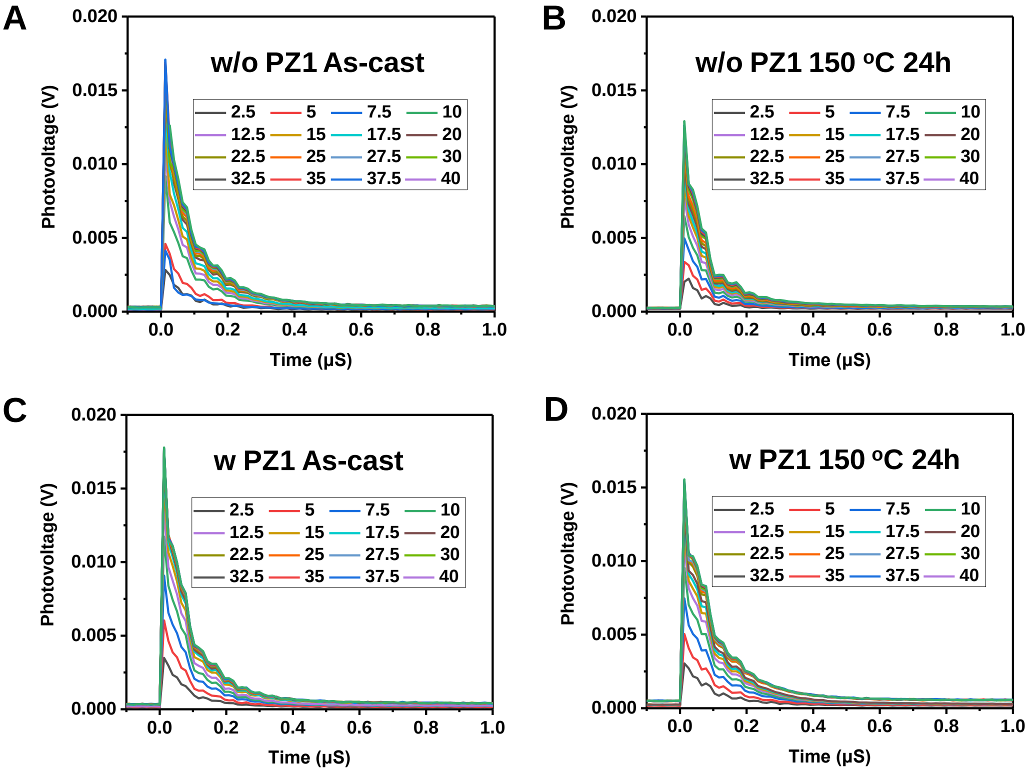

Supplementary Figure 22. measurements on PM6:BTTT-2Cl films (A) without heating and (B) with 150 °C for 24 h based devices for light intensities of 0.15 to 2.50 sun; TPV measurements on 1 wt% PZ1-incorporated PM6:BTTT-2Cl films (C) without heating and (D) with 150 °C for 24 h based devices.

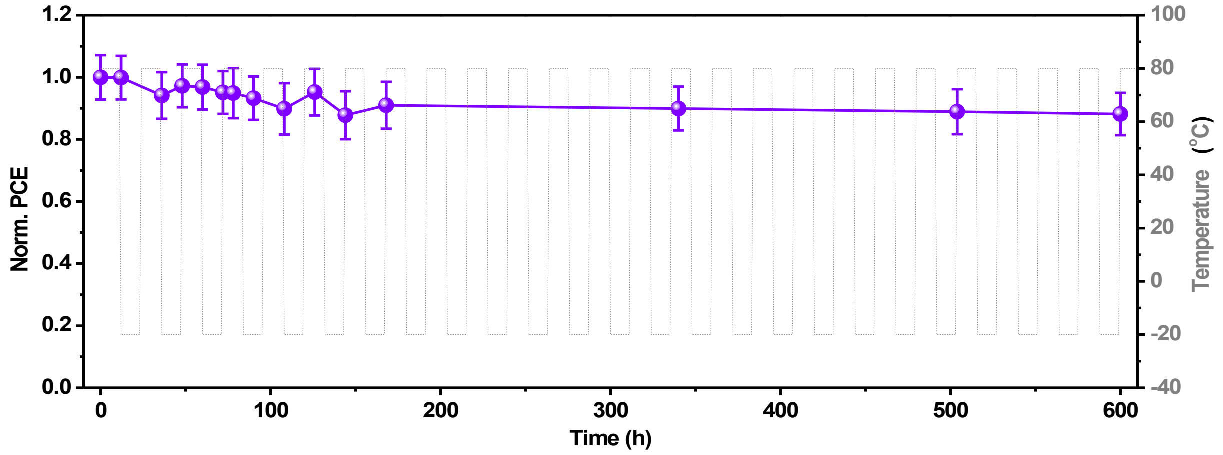

Supplementary Figure 23. Evolution of the device degradation of PZ1-doped PM6:BTTT-2Cl OSCs with an inverted structure under thermal cycling stress between -20 °C and 80 °C for 600 hours. All degradation data are averaged values of at least five solar cells.

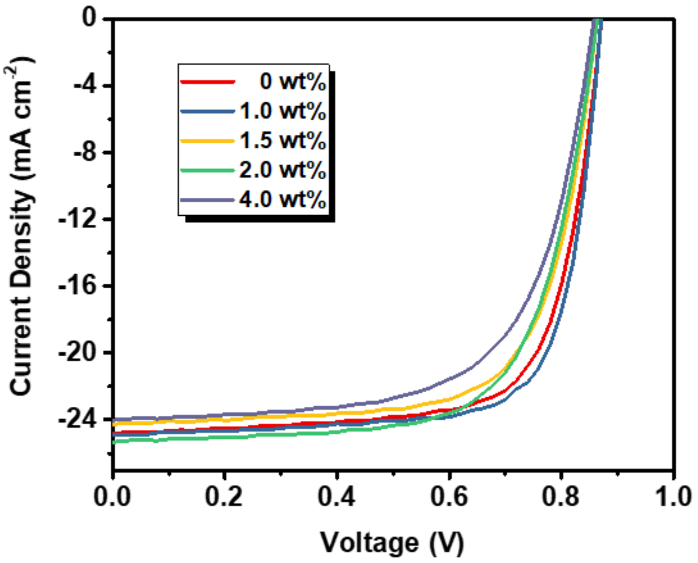

Supplementary Figure 24. *J-V* curves of the devices based on the PM6:Y6 blends without and with different PZ1 weight ratios.

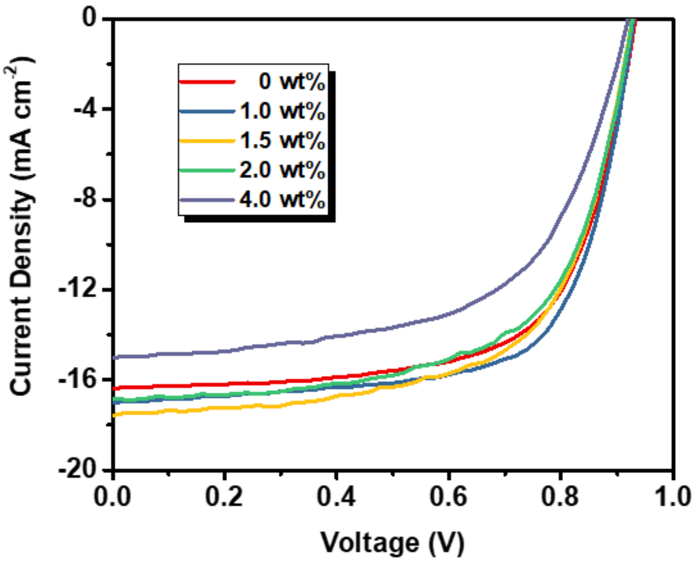

Supplementary Figure 25. *J-V* curves of the devices based on the J71:ITIC blends without and with different PZ1 weight ratios.

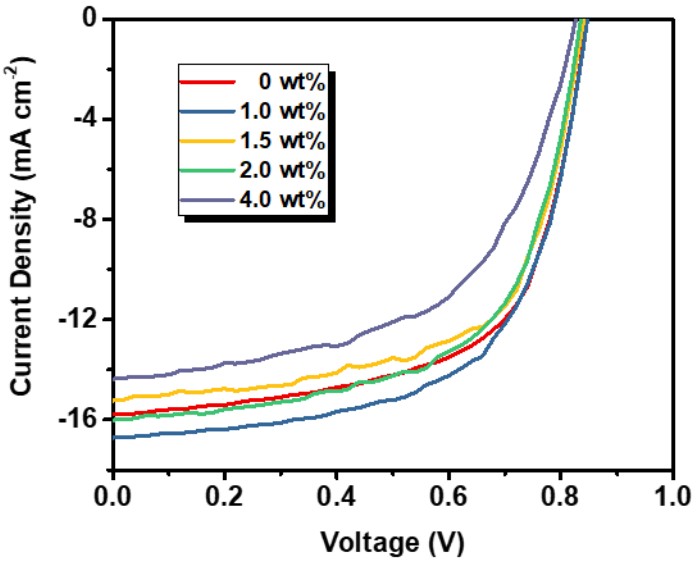

Supplementary Figure 26. *J-V* curves of the devices based on the PTB7-Th:$PC_{70}BM$ blends without and with different PZ1 weight ratios.

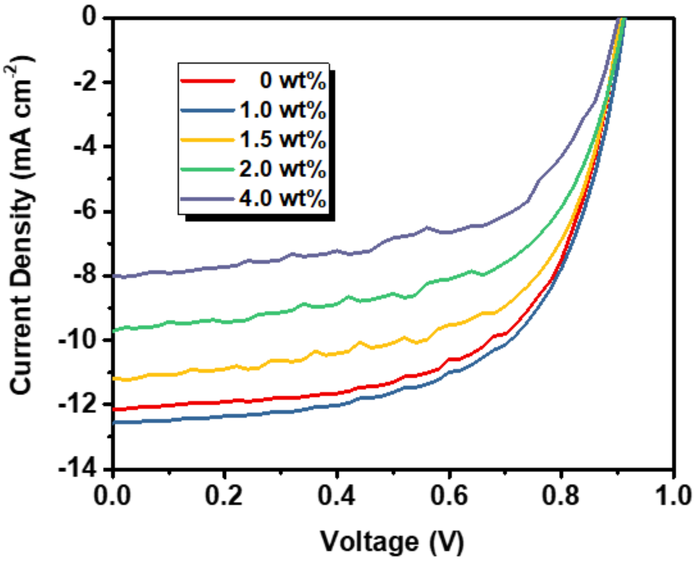

Supplementary Figure 27. *J-V* curves of the devices based on the BDT-3T-R:$PC_{70}BM$ blends without and with different PZ1 weight ratios.

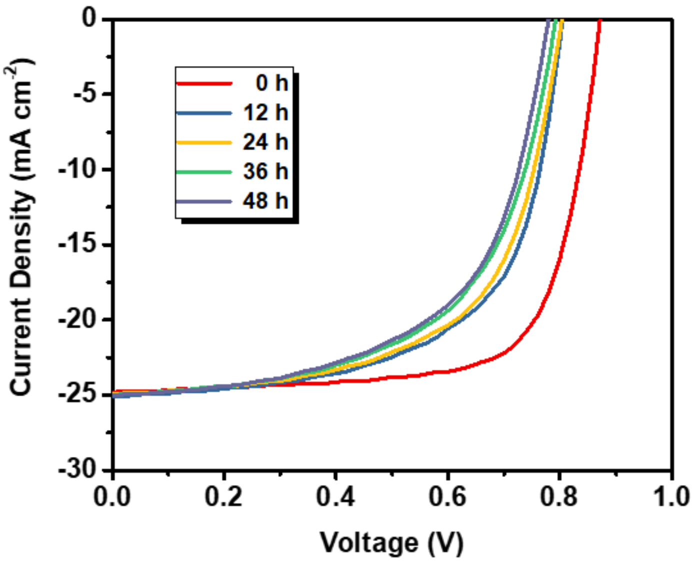

Supplementary Figure 28. *J-V* curves of PM6:Y6 blends without PZ1 heated under 150 ºC for 0, 12, 24, 36 and 48 hours.

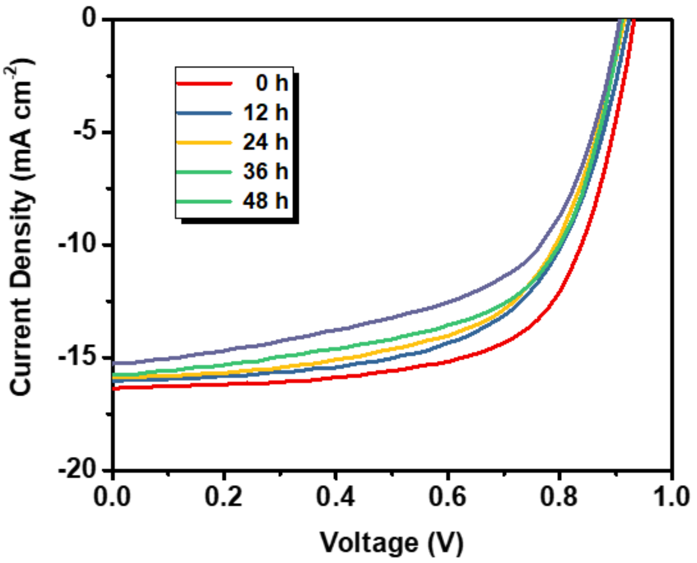

Supplementary Figure 29. *J-V* curves of J71:ITIC blends without PZ1 heated under 150 ºC for 0, 12, 24, 36 and 48 hours.

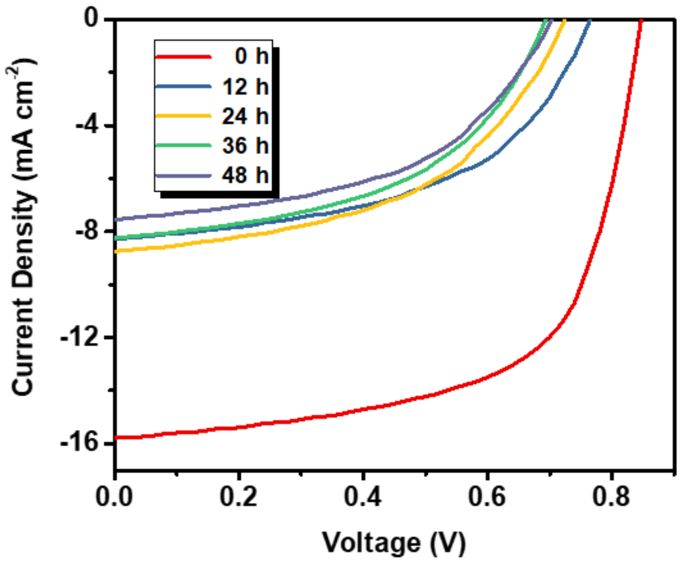

Supplementary Figure 30. *J-V* curves of PTB7-Th:$PC_{70}BM$ blends without PZ1 heated under 150ºC for 0, 12, 24, 36 and 48 hours.

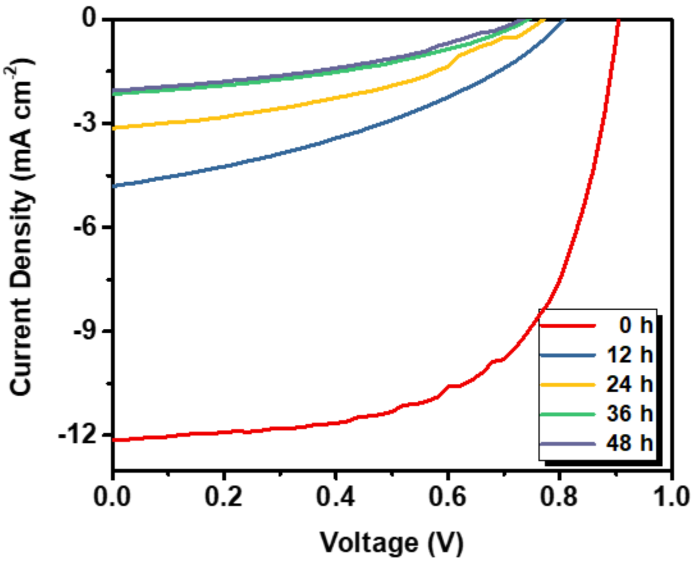

Supplementary Figure 31. *J-V* curves of BDT-3T-R:$PC_{70}BM$ blends without PZ1 heated under 150ºC for 0, 12, 24, 36 and 48 hours.

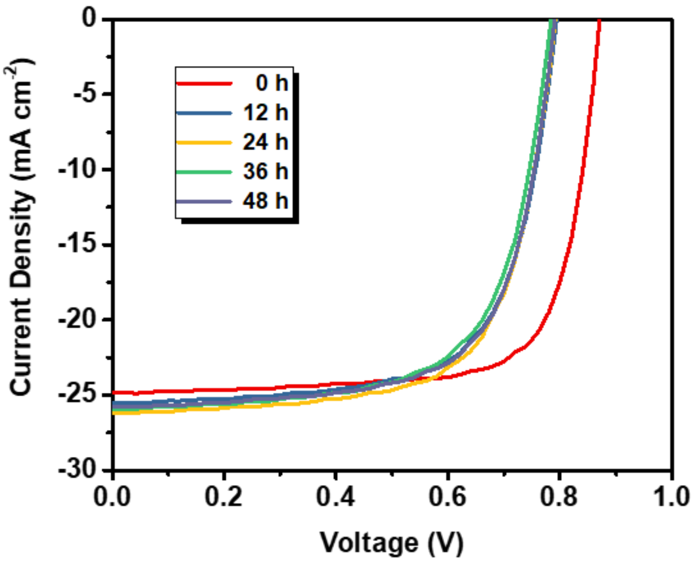

Supplementary Figure 32. *J-V* curves of PM6:Y6 blends with 1 wt% PZ1 heated under 150 ºC for 0, 12, 24, 36 and 48 hours.

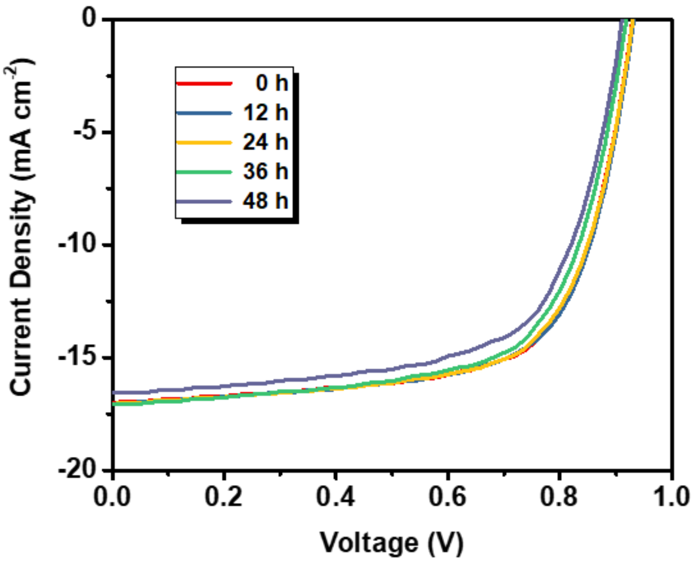

Supplementary Figure 33. *J-V* curves of J71:ITIC blends with 1 wt% PZ1 heated under 150 ºC for 0, 12, 24, 36 and 48 hours.

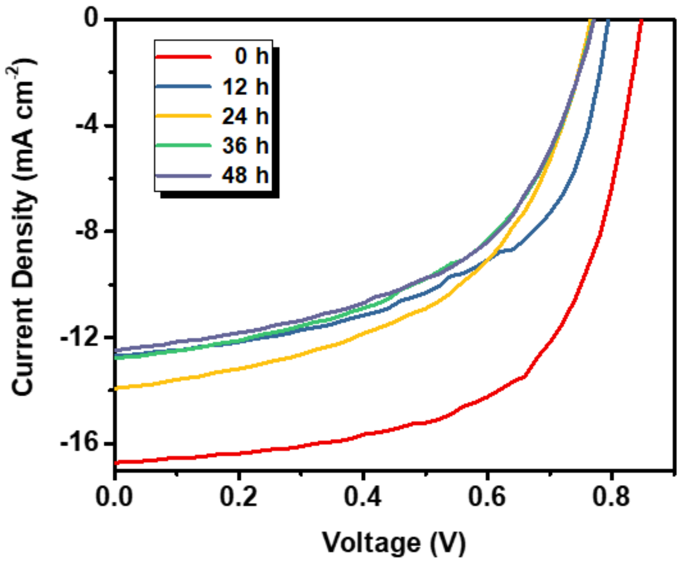

Supplementary Figure 34. *J-V* curves of PTB7-Th:$PC_{70}BM$ blends with 1 wt% PZ1 heated under 150 ºC for 0, 12, 24, 36 and 48 hours.

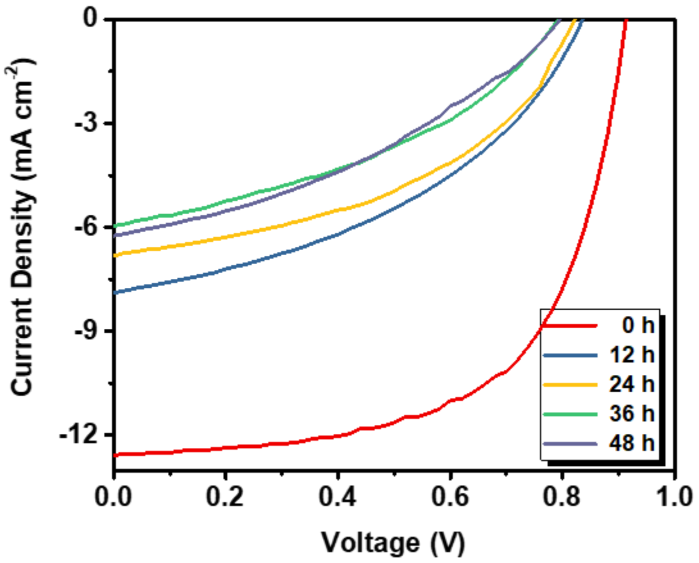

Supplementary Figure 35. *J-V* curves of BDT-3T-R:$PC_{70}BM$ blends with 1 wt% PZ1 heated under 150 ºC for 0, 12, 24, 36 and 48 hours.

Supplementary Tables

Supplementary Table 1. Summary of optical properties and electronic energy levels of BTTT-2Cl.

Solution $\lambda_{max}{}^{a}$ (nm)	Film $\lambda_{max}{}^{b}$ (nm)	$E_g{}^{opt,\,c}$ (eV)	HOMO[d] (eV)	LUMO[d] (eV)	$E_g{}^{cv}$ (eV)	HOMO[e] (eV)	LUMO[e] (eV)
751	825	1.36	-5.61	-3.98	1.63	-5.59	-3.59

[a] Measured in chloroform solution. [b] Cast from chloroform solution. [c] Bandgap estimated from the onset wavelength (λ_{edge}) of the optical absorption: $E_g{}^{opt} = 1240/\lambda_{edge}$. [d] Measured by electrochemical cyclic voltammetry. [e] Obtained by DFT calculation.

Supplementary Table 2. Photovoltaic parameters of PM6:BTTT-2Cl devices with different spin-coating speeds, measured under one sun illumination.

Speed [rpm]	*Voc* [V]	*Jsc* [mA cm^{-2}]	FF [%]	PCE (Avg.[a]) [%]
1000	0.90	23.92	60.54	13.04 (12.51)
1500	0.90	23.80	65.19	13.83 (13.30)
2000	0.90	22.76	66.56	13.71 (13.34)
2500	0.90	21.90	66.48	13.16 (12.45)
3000	0.90	20.29	66.23	12.10 (11.68)

[a] The values in bracket are the average PCE obtained from eight devices.

Supplementary Table 3. Photovoltaic parameters of PM6:BTTT-2Cl devices with different PZ1 weight ratios.

PZ1 [x wt%]	*Voc* [V]	*Jsc* [mA cm^{-2}]	FF [%]	PCE (Avg.[a]) [%]
0	0.90	23.80	65.19	13.83 (13.30)
0.5	0.90	23.79	65.53	13.85 (13.40)
1.0	0.90	24.58	67.90	15.10 (14.69)
1.5	0.90	23.28	66.77	14.05 (13.58)
2.0	0.90	22.98	60.16	12.47 (12.11)
4.0	0.90	22.14	58.64	11.68 (11.02)
8.0	0.90	22.95	55.03	11.35 (10.40)
16.0	0.90	22.20	40.01	8.02 (7.93)
32.0	0.90	19.85	42.71	7.61 (7.50)

[a] The values in bracket are the average PCE obtained from eight devices.

Supplementary Table 4. Photovoltaic parameters of PM6:BTTT-2Cl (1 wt% PZ1) devices with different spin-coating speeds.

Speed [rpm]	*Voc* [V]	*Jsc* [mA cm^{-2}]	FF [%]	PCE (Avg.[a]) [%]
1000	0.91	23.28	62.85	13.33 (12.98)
1300	0.91	23.89	63.51	13.84 (13.41)
1500	0.90	24.29	64.13	14.12 (13.86)
1800	0.90	24.30	66.55	14.62 (14.32)
2000	0.90	24.58	67.90	15.10 (14.69)
2500	0.90	23.27	67.52	14.18 (13.59)
3000	0.91	23.18	65.96	13.87 (13.25)
3500	0.91	21.68	65.68	12.92 (12.57)

[a] The values in bracket are the average PCE obtained from eight devices.

Supplementary Table 5. Summary of morphology formation kinetics data for PM6:BTTT-2Cl films without and with 1 wt% PZ1 at 150 °C for different time periods.

Name	Peak 1<br>PM6 (100)- in plane		Peak 1<br>BTTT-2Cl (010)- out of plane	
	FWHM ($Å^{-1}$)	CCL (nm)	FWHM ($Å^{-1}$)	CCL (nm)
PM6:BTTT-2Cl fresh	0.083	76.17	0.32	19.94
PM6:BTTT-2Cl 150 °C 5h	0.065	96.91	0.25	24.83
PM6:BTTT-2Cl 150 °C 24h	0.069	91.13	0.23	26.96
Blend with 1 wt% PZ1 fresh	0.095	66.17	0.29	21.68
Blend with 1 wt% PZ1 150 °C 5h	0.067	94.29	0.28	22.53
Blend with 1 wt% PZ1 150 °C 24h	0.063	99.12	0.26	23.88

Supplementary Table 6. Details of the thermal cycling test profiles of the blends under the different spaces.

Simulation condition	Moon	Mars	Low earth orbital Satellite
One cycle time	29 day	25 h	102 min
Ramp time	0.5 day	1h	3 min
Highest temperature	117 °C	35 °C	117 °C
Lowest temperature	-173 °C	-143 °C	-173 °C
Test cycle numbers	3	3	6

Supplementary Table 7. Photovoltaic parameters of PM6:Y6 devices with different PZ1 weight ratios.

PZ1 [x wt%]	*Voc* [V]	*Jsc* [mA cm^{-2}]	FF [%]	PCE (Avg.[a]) [%]
0	0.87	24.82	72.02	15.53 (15.23)
1.0	0.87	24.86	74.29	16.08 (15.70)
1.5	0.86	24.26	69.79	14.62 (14.47)
2.0	0.86	25.33	67.99	14.88 (14.44)
4.0	0.86	24.00	65.04	13.42 (13.17)

[a] The values in bracket are the average PCE obtained from eight devices.

Supplementary Table 8. Photovoltaic parameters of J71:ITIC devices with different PZ1 weight ratios.

PZ1 [x wt%]	*Voc* [V]	*Jsc* [mA cm^{-2}]	FF [%]	PCE (Avg.[a]) [%]
0	0.93	16.37	66.66	10.18 (10.03)
1.0	0.93	16.99	68.51	10.83 (10.40)
1.5	0.93	17.57	63.56	10.35 (10.09)
2.0	0.93	16.83	63.53	9.92 (9.54)
4.0	0.92	15.02	59.55	8.22 (7.95)

[a] The values in bracket are the average PCE obtained from eight devices.

Supplementary Table 9. Photovoltaic parameters of PTB7-Th:$PC_{70}BM$ devices with different PZ1 weight ratios.

PZ1 [x wt%]	V_{oc} [V]	J_{sc} [mA cm^{-2}]	FF [%]	PCE (Avg.[a]) [%]
0	0.85	15.79	63.06	8.43 (8.21)
1.0	0.85	16.71	62.73	8.88 (8.59)
1.5	0.84	15.23	63.35	8.11 (7.74)
2.0	0.84	16.00	61.08	8.17 (7.81)
4.0	0.83	14.37	56.06	6.65 (6.37)

[a] The values in bracket are the average PCE obtained from eight devices.

Supplementary Table 10. Photovoltaic parameters of BDT-3T-R:$PC_{70}BM$ devices with different PZ1 weight ratios.

PZ1 [x wt%]	*Voc* [V]	*Jsc* [mA cm^{-2}]	FF [%]	PCE (Avg.[a]) [%]
0	0.90	12.14	62.46	6.86 (6.52)
1.0	0.91	12.56	61.92	7.11 (6.99)
1.5	0.90	11.17	61.95	6.26 (6.10)
2.0	0.90	9.71	60.06	5.31 (4.98)
4.0	0.90	8.00	59.48	4.29 (4.02)

[a] The values in bracket are the average PCE obtained from eight devices.

Supplementary Table 11. Photovoltaic parameters of PM6:Y6 blends without PZ1 heated under 150 ºC for 0, 12, 24, 36 and 48 hours.

Time [h]	*Voc* [V]	*Jsc* [mA cm^{-2}]	FF [%]	PCE (Avg.[a]) [%]
0 h	0.87	24.82	72.02	15.53 (15.23)
12 h	0.81	25.08	62.11	12.60 (12.28)
24 h	0.80	24.95	61.35	12.26 (11.99)
36 h	0.79	25.00	58.70	11.58 (11.21)
48 h	0.78	25.03	58.40	11.40 (11.03)

[a] The values in bracket are the average PCE obtained from eight devices.

Supplementary Table 12. Photovoltaic parameters of J71:ITIC blends without PZ1 heated under 150 ºC for 0, 12, 24, 36 and 48 hours.

Time [h]	*Voc* [V]	*Jsc* [mA cm^{-2}]	FF [%]	PCE (Avg.[a]) [%]
0 h	0.93	16.37	66.66	10.18 (10.03)
12 h	0.92	16.06	62.06	9.22 (9.01)
24 h	0.92	15.89	61.96	9.00 (8.78)
36 h	0.91	15.78	61.54	8.85 (8.23)
48 h	0.91	15.28	57.74	8.00 (7.74)

[a] The values in bracket are the average PCE obtained from eight devices.

Supplementary Table 13. Photovoltaic parameters of PTB7-Th:$PC_{70}BM$ blends without PZ1 heated under 150 ºC for 0, 12, 24, 36 and 48 hours.

Time<br>[h]	*Voc*<br>[V]	*Jsc*<br>[mA cm^{-2}]	FF<br>[%]	PCE (Avg.[a])<br>[%]
0 h	0.85	15.79	63.06	8.43 (8.21)
12 h	0.76	8.28	50.80	3.21 (3.00)
24 h	0.72	8.75	49.33	3.17 (2.88)
36 h	0.69	8.25	49.66	2.84 (2.31)
48 h	0.70	7.55	49.76	2.65 (2.08)

[a] The values in bracket are the average PCE obtained from eight devices.

Supplementary Table 14. Photovoltaic parameters of BDT-3T-R:$PC_{70}BM$ blends without PZ1 heated under 150 °C for 0, 12, 24, 36 and 48 hours.

Time [h]	*Voc* [V]	*Jsc* [mA cm^{-2}]	FF [%]	PCE (Avg.[a]) [%]
0 h	0.90	12.14	62.46	6.86 (6.52)
12 h	0.81	4.81	37.28	1.45 (1.09)
24 h	0.77	3.14	39.71	0.96 (0.47)
36 h	0.74	2.15	39.42	0.63 (0.33)
48 h	0.73	2.05	37.94	0.56 (0.31)

[a] The values in bracket are the average PCE obtained from eight devices.

Supplementary Table 15. Photovoltaic parameters of PM6:Y6 blends with 1 wt% PZ1 heated under 150 ºC for 0, 12, 24, 36 and 48 hours.

Time<br>[h]	*Voc*<br>[V]	*Jsc*<br>[mA cm^{-2}]	FF<br>[%]	PCE (Avg.[a])<br>[%]
0 h	0.87	24.86	74.29	16.08 (15.70)
12 h	0.79	25.56	67.95	13.79 (13.61)
24 h	0.79	26.23	68.05	14.13 (13.87)
36 h	0.78	25.92	66.29	13.47 (13.32)
48 h	0.79	25.81	67.90	13.86 (13.57)

[a] The values in bracket are the average PCE obtained from eight devices.

Supplementary Table 16. Photovoltaic parameters of J71:ITIC blends with 1 wt% PZ1 heated under 150 ºC for 0, 12, 24, 36 and 48 hours.

Time [h]	*Voc* [V]	*Jsc* [mA cm^{-2}]	FF [%]	PCE (Avg.[a]) [%]
0 h	0.93	16.99	68.51	10.83 (10.40)
12 h	0.93	17.06	67.99	10.87 (10.54)
24 h	0.93	17.05	67.91	10.81 (10.37)
36 h	0.92	17.09	66.69	10.47 (10.12)
48 h	0.91	16.57	66.05	9.97 (9.85)

[a] The values in bracket are the average PCE obtained from eight devices.

Supplementary Table 17. Photovoltaic parameters of PTB7-Th:$PC_{70}BM$ blends with 1 wt% PZ1 heated under 150 ºC for 0, 12, 24, 36 and 48 hours.

Time [h]	*Voc* [V]	*Jsc* [mA cm^{-2}]	FF [%]	PCE (Avg.[a]) [%]
0 h	0.85	16.71	62.73	8.88 (8.59)
12 h	0.79	12.67	55.15	5.55 (5.03)
24 h	0.77	13.92	52.36	5.57 (4.74)
36 h	0.77	12.78	51.64	5.08 (4.66)
48 h	0.77	12.49	52.75	5.10 (4.72)

[a] The values in bracket are the average PCE obtained from eight devices.

Supplementary Table 18. Photovoltaic parameters of BDT-3T-R:$PC_{70}BM$ blends with 1 wt% PZ1 heated under 150 ºC for 0, 12, 24, 36 and 48 hours.

Time [h]	*Voc* [V]	*Jsc* [mA cm^{-2}]	FF [%]	PCE (Avg.[a]) [%]
0 h	0.91	12.56	61.92	7.11 (6.99)
12 h	0.84	7.89	41.85	2.76 (2.24)
24 h	0.82	6.82	44.58	2.50 (2.11)
36 h	0.79	5.95	38.80	1.83 (1.30)
48 h	0.79	6.23	36.65	1.82 (1.07)

[a] The values in bracket are the average PCE obtained from eight devices.

Supplementary Notes

Supplementary Note1. The synthesis of BTTT-2Cl.

(2,2′-((2Z,2′Z)-((12,13-bis(2-ethylhexyl)-3,9-diundecyl-12,13dihydro-[1,2,5]thiadiazolo [3,4-e]thieno[2″,3″:4′,5′]thieno[2′,3′:4,5]pyrrolo[3,2-g]thieno[2′,3′:4,5]thieno[3,2-b] indole-2,10-diyl)bis(methanylylidene))bis(2-chloro-6-oxo-5,6-dihydro-4H-cyclopenta [b]thiophene-4-ylidene)dimalononitrile) (BTTT-2Cl)

BTTT-CHO (140 mg, 0.136 mmol), CPTCN-Cl (129 mg, 0.55 mmol), chloroform (30 mL), and pyridine (1 mL) were added to a two-necked round-bottomed flask. The mixture was deoxygenated with nitrogen for 30 min and then refluxed for 24 h. After cooling to room temperature, the mixture was poured into methanol (200 mL) and filtered. The residue was purified by column chromatography on silica gel using petroleum ether/dichloromethane (1:2) as eluent, yielding a dark blue solid (148 mg, 75%). ^{1}H NMR ($CDCl_3$, 400 MHz): δ [ppm]: 9.07 (s, 2H), 8.17 (s, 2H), 4.78-4.74 (m, 4H), 3.22 (t, J = 8.0 Hz, 4H), 2.21-2.12 (m, 2H), 1.93-1.80 (m, 4H), 1.50-1.46 (m, 4H), 1.26-1.16 (m, 32H), 1.14-0.92 (m, 12H), 0.84-0.78 (m, 6H), 0.75-0.60 (m, 12H).^{13}C NMR (100 MHz, $CDCl_3$) δ [ppm]: 180.34, 155.62, 153.91, 147.45, 145.32, 141.45, 138.03, 136.04, 133.67, 133.55, 131.55, 126.53, 125.24, 115.12, 114.96, 113.70, 66.34, 55.62, 31.91, 29.82, 29.64, 29.62, 29.52, 29.47, 29.34, 22.69, 14.14, 13.75. MALDI-TOF MS: calcd for [$C_{78}H_{84}Cl_2N_8O_2S_7$] ($M^+$): Calc. m/z: 1458.4129, found: 1459.4130.

Supplementary Note 2. The analysis of bimolecular and trap-assisted recombination.

We studied the bimolecular recombination mechanism by measuring the photocurrent (J_{sc}) at various light intensities (I) from 100 to 1 mA cm^{-2}, as shown in Supplementary Figure 17A. A power-law dependence of J_{sc} upon illumination intensity, which could effectively quantify the bimolecular recombination mechanisms, can be expressed as $J_{sc} = \beta(I)^{\alpha}$, where β is a constant and α is the exponential factor. The best fit for the data is obtained when the value α is close to unity, which indicates negligible bimolecular recombination during sweep-out. The slop (α) of the undoped device is 0.996, whereas that of doped device is 1.001, suggesting that the bimolecular recombination are not prominent in these two types of devices as described.

Multiple studies have demonstrated that the light intensity dependence of the V_{oc} can directly provide insight into the role of trap-assisted recombination versus 2nd order recombination at the open circuit condition. The V_{oc} and light intensity (I) can be correlated by the expression of $V_{OC} = \frac{E_{\text{gap}}}{q} - \frac{kT}{q}\ln[\frac{(1-P)\gamma N_C^2}{PG}]$, where E_{gap} is the energy difference between the highest occupied molecular orbital (HOMO) of the electron donor and the lowest unoccupied molecular orbital (LUMO) of the electron acceptor, q is the elementary charge, k is the Boltzman constant, T is temperature in Kelvin, P is the dissociation probability of the electron-hole pairs into free carriers, γ the recombination constant, N_c the density of states in the conduction band, and G the generation rate of electron-hole pairs. Following the rules, the formula predicts a slope $S = (kT/q)$ of the V_{oc} versus the natural logarithm of the incident light intensity. This implies that the slope of V_{oc} versus ln(I) is equal to kT/q for bimolecular recombination. When the additional mechanism of Schockley-Real-Hall (SRH) or trap-assisted recombination is involved, a stronger dependency of V_{oc} on the light intensity is observed and in this case, the slope of V_{oc} versus ln(I) is equal to 2 kT/q. Figure S17B shows the V_{oc} versus light intensity relationship for devices based on undoped and doped active layers. The doped device exhibits a logarithmic dependence on light

intensity with a slope of 1.36 *kT/q*. While the slope yields 1.44 *kT/q* for the undoped device. As compared to the doped devices, the slightly stronger dependence of V_{oc} on light intensity implies that carrier dynamics at open circuit in BHJ device is slightly governed by a combination of trap-assisted (SRH) type and bimolecular recombination. The use of doped layer reduces the trap-assisted recombination as seen from a slope of 1.36 *kT/q*. We speculate that 1 wt% PZ1-doped morphology might not only reduce the number of trapping defects in bulk.

Supplementary References

1 Fan, Q. *et al.* Synergistic effect of fluorination on both donor and acceptor materials for high performance non-fullerene polymer solar cells with 13.5% efficiency. *Sci. China: Chem.* **61**, 531-537 (2018).

2 Bin, H. *et al.* 11.4% Efficiency non-fullerene polymer solar cells with trialkylsilyl substituted 2D-conjugated polymer as donor. *Nat. Commun.* **7**, 13651 (2016).

3 Badgujar, S. *et al.* Highly efficient and thermally stable fullerene-free organic solar cells based on a small molecule donor and acceptor. *J. Mater. Chem. A* **4**, 16335-16340 (2016).

4 Yuan, J. *et al.* Single-Junction Organic Solar Cell with over 15% Efficiency Using Fused-Ring Acceptor with Electron-Deficient Core. *Joule* **3**, 1140-1151 (2019).
