# Supplementary material for: Simultaneous enhanced efficiency and thermal stability in organic solar cells from a polymer acceptor additive
Source: Nat Commun. 2020 Mar 5;11:1218. doi: 10.1038/s41467-020-14926-5 (PMC7057953; doi:10.1038/s41467-020-14926-5)
Supplement: Supplementary file 3 — Reporting Summary [file 41467_2020_14926_MOESM3_ESM.pdf]

nature research

Corresponding author(s): Jie Min

Last updated by author(s): Feb 3, 2020

Reporting Summary

Nature Research wishes to improve the reproducibility of the work that we publish. This form provides structure for consistency and transparency in reporting. For further information on Nature Research policies, seeAuthors & Referees and theEditorial Policy Checklist .

Statistics

For all statistical analyses, confirm that the following items are present in the figure legend, table legend, main text, or Methods section.

n/a	Confirmed	
☐	☒	The exact sample size (*n*) for each experimental group/condition, given as a discrete number and unit of measurement
☐	☒	A statement on whether measurements were taken from distinct samples or whether the same sample was measured repeatedly
☐	☒	The statistical test(s) used AND whether they are one- or two-sided<br>*Only common tests should be described solely by name; describe more complex techniques in the Methods section.*
☒	☐	A description of all covariates tested
☐	☒	A description of any assumptions or corrections, such as tests of normality and adjustment for multiple comparisons
☐	☒	A full description of the statistical parameters including central tendency (e.g. means) or other basic estimates (e.g. regression coefficient) AND variation (e.g. standard deviation) or associated estimates of uncertainty (e.g. confidence intervals)
☒	☐	For null hypothesis testing, the test statistic (e.g. *F*, *t*, *r*) with confidence intervals, effect sizes, degrees of freedom and *P* value noted<br>*Give P values as exact values whenever suitable.*
☒	☐	For Bayesian analysis, information on the choice of priors and Markov chain Monte Carlo settings
☒	☐	For hierarchical and complex designs, identification of the appropriate level for tests and full reporting of outcomes
☒	☐	Estimates of effect sizes (e.g. Cohen's *d*, Pearson's *r*), indicating how they were calculated

Our web collection on statistics for biologists contains articles on many of the points above.

Software and code

Policy information about availability of computer code

Data collection	We used excel and origin 9.0 software for data collection.
Data analysis	We also used excel and origin 9.0 software for data analysis.

For manuscripts utilizing custom algorithms or software that are central to the research but not yet described in published literature, software must be made available to editors/reviewers. We strongly encourage code deposition in a community repository (e.g. GitHub). See the Nature Research guidelines for submitting code & software for further information.

Data

Policy information about availability of data

All manuscripts must include a data availability statement. This statement should provide the following information, where applicable:

- Accession codes, unique identifiers, or web links for publicly available datasets
- A list of figures that have associated raw data
- A description of any restrictions on data availability

The relevant data are available from the authors upon reasonable request.

Field-specific reporting

Please select the one below that is the best fit for your research. If you are not sure, read the appropriate sections before making your selection.

☐ Life sciences ☐ Behavioural & social sciences ☒ Ecological, evolutionary & environmental sciences

For a reference copy of the document with all sections, see nature.com/documents/nr-reporting-summary-flat.pdf

Ecological, evolutionary & environmental sciences study design

All studies must disclose on these points even when the disclosure is negative.

Study description	In this study, we report a PZ1-doping general strategy and develop a new thermally stable bulk heterojunction (BHJ) blend, which composed of the designed and synthesized non-fullerene acceptor BTTT-2Cl and the commercially available polymer donor PM6. Supported by statistics of devices degradation data analysis, and morphological characteristics and physical mechanisms study, the PM6:BTTT-2Cl blends with 1 wt% PZ1 polymer acceptors show longer lifetime with nearly keeping their efficiency (t = 800 h) under accelerated heating test at 150 oC, while power conversion efficiency (PCE) of normal blends without PZ1 drop dramatically up to 48.4% of its initial value (a PCE of 13.80%) in 24 h.
Research sample	We used a new thermally stable bulk heterojunction (BHJ) blend, which composed of the designed and synthesized non-fullerene acceptor BTTT-2Cl and the commercially available polymer donor PM6. In addition, a polymer acceptor PZ1 was introduced as the solid additives. The corresponding data can be found in source data file.
Sampling strategy	In this study, we repeated the device performance for several times. And the photovoltaic parameters as well as their trends under thermal stress can also be repeated. In addition, the relevant values and data provided in the article and supporting information files are achieved from eight independent devices.
Data collection	All of the data were measured and collected by Wenyan Yang, who is a master student. In addition, the original data are also stored in our public computer.
Timing and spatial scale	This wok was begun in April 2019. In addition, this project was due to end in November 2019. All of the data are collected in this time period. For the detailed data collection time, the corresponding explanation can be found in the main text and experimental section.
Data exclusions	No data were excluded from the analyses.
Reproducibility	all attempts to repeat the experimental were successful.
Randomization	This is not relevant to this study. All of materials come from the other labs (e.g. Prof. Chuluo Yang's group) and relevant companies.
Blinding	Blinding was not possible. All of the tables and figures are tested and made by ourselves. The data in this article were not selected in great detail.

Did the study involve field work? ☐ Yes ☒ No

Reporting for specific materials, systems and methods

We require information from authors about some types of materials, experimental systems and methods used in many studies. Here, indicate whether each material, system or method listed is relevant to your study. If you are not sure if a list item applies to your research, read the appropriate section before selecting a response.

Materials & experimental systems

n/a	Involved in the study
☒	☐ Antibodies
☒	☐ Eukaryotic cell lines
☒	☐ Palaeontology
☒	☐ Animals and other organisms
☒	☐ Human research participants
☒	☐ Clinical data

Methods

n/a	Involved in the study
☒	☐ ChIP-seq
☒	☐ Flow cytometry
☒	☐ MRI-based neuroimaging
