# Supplementary material for: Simultaneous enhanced efficiency and thermal stability in organic solar cells from a polymer acceptor additive
Source: Nat Commun. 2020 Mar 5;11:1218. doi: 10.1038/s41467-020-14926-5 (PMC7057953; doi:10.1038/s41467-020-14926-5)
Supplement: Supplementary file 4 — Solar Cells Reporting Summary [file 41467_2020_14926_MOESM4_ESM.pdf]

nature research

Corresponding author(s): Jie Min

Solar Cells Reporting Summary

Nature Research wishes to improve the reproducibility of the work that we publish. This form is intended for publication with all accepted papers reporting the characterization of photovoltaic devices and provides structure for consistency and transparency in reporting. Some list items might not apply to an individual manuscript, but all fields must be completed for clarity.

For further information on Nature Research policies, including our data availability policy, see Authors & Referees.

ü Experimental design

Please check: are the following details reported in the manuscript?

1. Dimensions

Item	Yes/No	Details
Area of the tested solar cells	[x] Yes [ ] No	The active area of the standard solar cell fabricated in our lab is 4 mm2. Details are given in the Experimental Section.
Method used to determine the device area	[x] Yes [ ] No	For the solar cells fabricated, the electronic active area of the cell, which was 4 mm2, is defined by the overlap of the ITO electrode and metal electrode. As we clearly describe in the manuscript, we used the Jsc measured on the properly masked and mismatch corrected standard area cell to self-calibrate the Jsc and equivalent active area on the small area cell. A full description is given in the Supplementary Information.

2. Current-voltage characterization

Item	Yes/No	Details
Current density-voltage (J-V) plots in both forward and backward direction	[ ] Yes [x] No	In general, organic solar cells show the same current density-voltage curves in both forward and backward direction. Thus, we only scan the solar cells in forward direction.
Voltage scan conditions<br>*For instance: scan direction, speed, dwell times*	[x] Yes [ ] No	We scan the solar cells in the range of -0.5-1.2V with a rate of 20 mV s-1.
Test environment<br>*For instance: characterization temperature, in air or in glove box*	[x] Yes [ ] No	The measurements were carried out in nitrogen glovebox at room temperature.
Protocol for preconditioning of the device before its characterization	[x] Yes [ ] No	For the devices measured under one sun illumination, no preconditioning was applied.
Stability of the J-V characteristic<br>*Verified with time evolution of the maximum power point or with the photocurrent at maximum power point; see ref. 7 for details.*	[x] Yes [ ] No	We tested the thermal stability of OSCs stored in glovebox. However, we did not provide the stability data of OSCs at maximum power point in this work.

3. Hysteresis or any other unusual behaviour

Item	Yes/No	Details
Description of the unusual behaviour observed during the characterization	[ ] Yes [x] No	There is no unusual behaviour observed during the characterization, i.e., hysteresis.
Related experimental data	[ ] Yes [x] No	We did not find the unusual behaviour.

4. Efficiency

Item	Yes/No	Details
External quantum efficiency (EQE) or incident photons to current efficiency (IPCE)	[x] Yes [ ] No	We provided the EQE data under the standard reference spectrum which is comparable with the simulator. An Enli Solar simulator was used as light source and the light intensity was calibrated with a standard single-crystal Si solar cell made by Enli Technology CO., Ltd., Taiwan, calibrated by The National Institute of Metrology (NIM) of China. We show details in the Experimental Section.
A comparison between the integrated response under the standard reference spectrum and the response measure under the simulator	[x] Yes [ ] No	The difference between the integrated current from EQE and the short-circuit current from the JV curve measured under AM 1.5G solar simulator is within 5% difference which is within the accuracy confidence of the measurements. We give full details in the main text and Experimental Section.
For tandem solar cells, the bias illumination and bias voltage used for each subcell	[ ] Yes [x] No	We did not make the tandem solar cells in this article.

5. Calibration

Item	Yes/No	Response
Light source and reference cell or sensor used for the characterization	[x] Yes [ ] No	An Enli Solar simulator was used as light source and the light intensity was calibrated with a standard single-crystal Si solar cell made by Enli Technology CO., Ltd., Taiwan, calibrated by The National Institute of Metrology (NIM) of China. We show details in the Experimental Section. The J-V curves are measured under simulated AM 1.5 sunlight at 100 mW cm-2 irradiance generated by a xenon lamp.
Confirmation that the reference cell was calibrated and certified	[x] Yes [ ] No	A standard single-crystal Si solar cell as the reference cell was made by Enli Technology CO., Ltd., and calibrated by The National Institute of Metrology (NIM) of China. This reference cell was used to calibrate the variable intensity under concentrated sun light, with an individual mismatch factor estimation applied for every intensity.
Calculation of spectral mismatch between the reference cell and the devices under test	[x] Yes [ ] No	The Enli Technology CO., Ltd. did the calculation of spectral mismatch between the reference cell and the devices. The necessary description was provided in the Supplementary Information.

6. Mask/aperture

Item	Yes/No	Response
Size of the mask/aperture used during testing	[ ] Yes [x] No	We did not use the mask/aperture during testing.
Variation of the measured short-circuit current density with the mask/aperture area	[ ] Yes [x] No	We did not use the mask/aperture during testing for measuring short-circuit current density.

7. Performance certification

Item	Yes/No	Response
Identity of the independent certification laboratory that confirmed the photovoltaic performance	[ ] Yes [x] No	We didn't do the independent certification. Because the device efficiency achieved in this article is not so high. In addition, our work is mainly focus on the thermal stability issue. The performance certification is not very important in this work.
A copy of any certificate(s)<br>*Provide in Supplementary Information*	[ ] Yes [x] No	We didn't do the performance certification via the independent certification laboratory.

8. Statistics

Item	Yes/No	Response
Number of solar cells tested	[x] Yes [ ] No	We have tested multiple tens of cells in our lab. In Table 1, The average PCEs were obtained from eight independent devices. In Figure 2 and Figure 4, the thermal stability of corresponding photovoltaic systems were evaluated by eight independent data.
Statistical analysis of the device performance	[x] Yes [ ] No	We give statistical data of the device performance in the Source Data file.

9. Long-term stability analysis

Item	Yes/No	Response
Type of analysis, bias conditions and environmental conditions<br>*For instance: illumination type, temperature, atmosphere humidity, encapsulation method, preconditioning temperature*	[x] Yes [ ] No	All measurements were performed on unencapsulated cells in glovebox at different temperatures as shown in Figure 2D and Figure 4 as well as Figure S20.
